# Research Progress on Rolling Circle Amplification (RCA)-Based Biomedical Sensing

**DOI:** 10.3390/ph11020035

**Published:** 2018-04-21

**Authors:** Lide Gu, Wanli Yan, Le Liu, Shujun Wang, Xu Zhang, Mingsheng Lyu

**Affiliations:** 1 College of Marine Life and Fisheries, Huahai Institute of Technology, Lianyungang 222005, China; gu_lide@163.com (L.G.); yanwanli003@163.com (W.Y.); liulezz3@163.com (L.L.); 2 Marine Resources Development Institute of Jiangsu, Lianyungang 222005, China; shujunwang86@163.com; 3 Co-Innovation Center of Jiangsu Marine Bio-industry Technology, Huaihai Institute of Technology, Lianyungang 222005, China; xu_zhang@cbu.ca; 4 Verschuren Centre for Sustainability in Energy & the Environment, Cape Breton University, Sydney, NS B1P 6L2, Canada

**Keywords:** rolling circle amplification (RCA), biosensor, clinical diagnostics, cancer

## Abstract

Enhancing the limit of detection (LOD) is significant for crucial diseases. Cancer development could take more than 10 years, from one mutant cell to a visible tumor. Early diagnosis facilitates more effective treatment and leads to higher survival rate for cancer patients. Rolling circle amplification (RCA) is a simple and efficient isothermal enzymatic process that utilizes nuclease to generate long single stranded DNA (ssDNA) or RNA. The functional nucleic acid unit (aptamer, DNAzyme) could be replicated hundreds of times in a short period, and a lower LOD could be achieved if those units are combined with an enzymatic reaction, Surface Plasmon Resonance, electrochemical, or fluorescence detection, and other different kinds of biosensor. Multifarious RCA-based platforms have been developed to detect a variety of targets including DNA, RNA, SNP, proteins, pathogens, cytokines, micromolecules, and diseased cells. In this review, improvements in using the RCA technique for medical biosensors and biomedical applications were summarized and future trends in related research fields described.

## 1. Introduction

Rolling circle amplification (RCA) is a commonly used research tool in molecular biology, materials science, and medicine [1,2,3]. Since its discovery at the end of 20th century, the applications of RCA have been increasing consistently with the development of science and technology [4]. RCA is an isothermal enzymatic process that uses DNA or RNA polymerases (e.g., Φ29 DNA polymerase) to produce single stranded DNA (ssDNA) or RNA molecules which are a connection in series of complementary units of a template. The process is simple and efficient [5,6,7,8,9,10]. An RCA reaction contains four parts: (1) a DNA polymerase and homologous buffer; (2) a relatively short DNA or RNA primer; (3) a circle template; and (4) deoxynucleotide triphosphates (dNTPs). In RCA, nucleotides (nt) were added continuously to a primer annealed to a circular template by polymerase, which produces a long ssDNA with hundreds to thousands of repeat units. It should also be noted that RCA products possess multiple repetitive sequence units, which correspond to the circular DNA template; therefore, they can be processed through modification of the template. By transforming the substrate, the DNA products can be customized to include functional sequences, including DNA aptamers [5,6], spacer domains [6], DNAzymes [7,8,9,10], and restriction enzyme sites [11,12,13]. Furthermore, multifunctional materials with diverse properties can also be made via hybridizing RCA products with complementary oligonucleotides tethered to functional moieties. These include fluorescent dyes, electrochemical tags, biotin, antibodies, enzymes and nanoparticles [14,15,16,17], which can then be used for sensitive detection, biorecognition, immunosensing and bioimaging.

Recently, RCA had been utilized to study and develop sensitive detection methods for DNA [9,11,18,19,20,21,22,23,24], RNA [25,26,27], DNA methylation [28,29], single nucleotide polymorphisms (SNP) [30,31,32], small molecules [7,33,34], target proteins [10,35], and cancer cells [6,36,37]. The circular templates are designed in RCA so that a single binding event can be amplified over a thousand-fold. The signal from a single binding event can be amplified in an exponential manner. RCA is ideal for the required ultrasensitive detection [38]. The feature is especially useful for diagnostics. The isothermal nature of RCA provides new possibilities for targeted therapy compared to other methods, such as polymerase chain reaction (PCR), which uses intricate and expensive apparatus (e.g., temperature gradient). Owing to these inherent excellent properties, multifarious, RCA-based platforms have been applied to test various types of targets, such as DNA, RNA, SNP, proteins, pathogens, cytokines, and tumor cells. In this review, improvements in the use of the RCA technique for medical biosensors and biomedical applications will be summarized and future trends in related research fields will be examined. 

## 2. Enzyme-Aided RCA Biosensor

Organisms contain a variety of enzymes, and changes in these enzymes provide a lot of important information, particularly for medical diagnoses. The study of these enzymes can help not only with the diagnosis, but also with the treatment of various diseases. Because RCA has many advantages (such as simplicity, efficiency, tunability), it thus plays an important role in enzyme research. RCA-mediated enzymatic reaction catalyzed amplification provides dual-amplification for ultrasensitive detection of analytes due to the excellent catalytic nature of some enzymes and efficient amplification of RCA. Some DNAzymes can combine with RCA to detect the sensitivity and specificity of other enzymes, such as DNA ligase and polynucleotide kinase/phosphatase (PNKP). DNA ligase, an extremely important member of the enzyme family, which can connect the 3′-hydroxyl and 5′-phosphoryl termini of fractured DNA to form phosphodiester bonds, and plays indispensable roles in DNA replication, repair, and recombination [39,40,41]. Recent clinical researches revealed that the activity level of DNA ligase is connected with the pathogenesis of cancer. Inhibiting the activity of DNA ligase can decrease cancer cell proliferation and metastasis, and immensely augment the sensitivity of cancer cells to anticancer drugs [42,43]. Polynucleotide kinase/phosphatase (PNKP) is a bifunctional enzyme with 5′-kinase and 3′-phosphatase activities; once the DNA strand is broken, its end will produce 5′-phosphate and 3′-hydroxyl groups, thus allowing some specific proteins to replace lost nucleotides and regroup broken strands [44]. PNKP also plays an important role in nucleic acid metabolism and DNA repair during strand interruption [45]. Inhibition of PNKP can also promote the sensitivity of human tumors to γ-radiation [46], which could be useful for enhancing the efficacy of existing cancer treatments [45].

**Figure 1:**
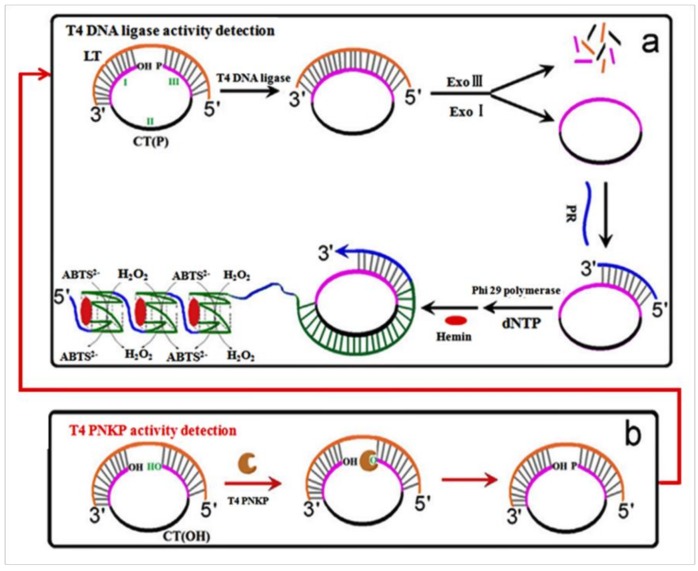
Schematic diagram of G-quadruplex DNAzyme-based DNA ligase sensor. (**a**) The CT composed of three parts. Part I and III could hybridize with LT and form a split ring. Part II was a C-rich area where G-rich sequences are generated to form G-quadruplex structures after RCA. The split was repaired when T4 DNA ligase was introduced; then, LT was excised by Exo I and Exo III After annealing, PR was adhered to the circular template and activated RCA with Phi29 polymerase. Numerous G-rich sequences will be produced to fold into G-quadruplex units and bind hemin to form catalytic G-quadruplex DNAzymes, which can catalyze the oxidation of ABTS^2−^ by H_2_O_2_ to ABTS, enhancing the absorption signal. (**b**) When the PNKP-triggered 5′-phosphroylation step was added to the substrate DNA, a PNKP sensor was easily designed based on the above sensing strategy. (LT, linear template; CT, circular template; ABTS, 2,2′-azino-bis(3-ethylbenzothiazoline-6-sulfonic acid)) [47].

In order to ascertain the activity of a DNA ligase, a G-quadruplex DNAzyme-based DNA ligase sensor was developed by Jiang et al. [47]. In their design, the oligonucleotide probes did not need to be labeled due to the use of G-quadruplex DNAzyme; hence, label-free detection was possible. The amplification of RCA will generate multimeric G-quadruplexes containing thousands of G-quadruplex units and revealed highly active hemin-binding sites, resulting in great improvements in sensor sensitivity. This sensor enabled the specific detection of T4 DNA ligase at a level as low as 1.9 U/μL. When the PNKP-triggered 5′-phosphroylation step was added to the substrate DNA, a PNKP sensor was easily designed on the basis of the above sensing strategy (Figure 1), which allowed specific detection of T4 PNKP at a limit of 1.8 U/μL.

This procedure is quite simple. A visual result will be obtained through a colorimetric reaction. It is easy, efficient and accurate. Meantime, the concept is also suitable for analysis of other enzymes.

## 3. AuNP-RCA Biosensor for Multiple Pathogens Detection

**Figure 2:**
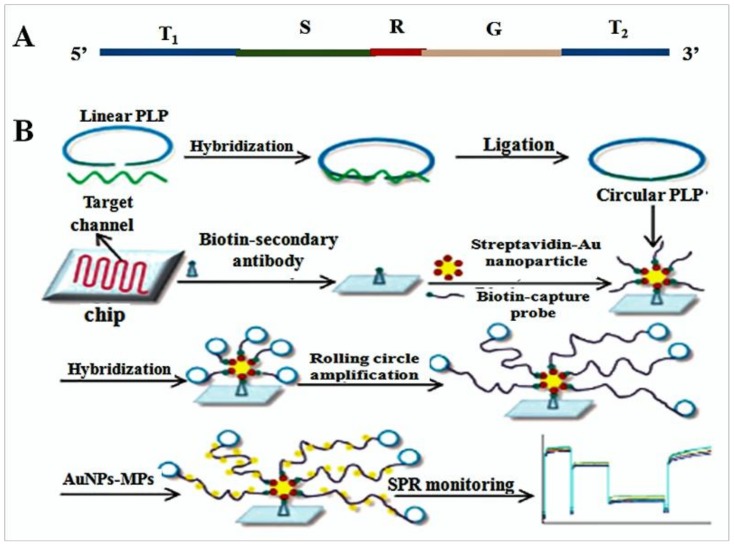
Schematic diagram of AuNP-RCA sensor for multiple pathogens detection. (**A**) The PLP was designed as 5 regions which contain target-complementary sequences at the 5′ and 3′ ends (T1 and T2); the CPs hybridizes with a sequence-specific region (S); a Hpal restriction endonuclease digestion site (R) and the AuNP-MP binding sequences: a general region (G). (**B**) The scheme of detection process. (1) Linear PLP and target sequences were hybridized and linked to form circular template. (2) Immobilizing biotin-secondary antibody on the chip surface and incubated with streptavidin-Au nanoparticles and biotin-capture probe. (3) Circular PLP was added and hybridized with CPs to activate the RCA reaction, which can produce abundant binding sites for AuNP-MP and output signal of SPR [56].

Many diseases are related to the invasion of pathogens. Studying the characteristics of pathogenic bacteria may help in developing treatments for these diseases. A fast and accurate definitive diagnosis of specific pathogenic bacteria is extremely important in clinical diagnostics. There are many effective techniques that are traditionally used to identify pathogens; however, these approaches can only confirm a single pathogen per assay [48,49,50,51,52,53]. Therefore, developing an all-purpose detection method for concurrent recognition of multiple pathogens is vitally important for improving detection efficacy. SPR biosensors may afford an opportunity to detect diverse pathogens. SPR is an optical detection technology, which utilizes the refraction and reflection of light. In this process, DNA probes are immobilized on the sensor surface and the complex of target molecules are introduced to traverse these clusters continuously [54]. Due to the sensitivity of the optical device, the detection process can be operated and monitored in real-time; therefore, SPR biosensors offer simple, sensitive, and on-site analysis [55]. Shi et al. [56] made a characteristic biosensor for the detection of pathogenic bacteria. In their study, an SPR DNA biosensor with a gold (Au) nanoparticle surface, based on RCA, was developed for isothermal recognition of DNA. The sensor contained a specific padlock probe (PLP) and a capture probe (CP), which were connected by biotin, and an Au nanoparticle-modified probe, the products of RCA were hybridized with them (Figure 2). The CP was fixed on AuNP in order to enhance the binding and hybridizing abilities of the biosensor. The linear PLP was cyclized by ligase after hybridizing with the pathogenic bacteria-specific sequences in 16S rDNA and the circular product was fully complementary with the CP. The corresponding channel on the chip surface distinguished every target gene DNA during the recognition process. The in situ solid-phase RCA reaction was then initiated by the immobilized CPs, which were regarded as primers, and produced long single-stranded DNA. The SPR angle changed with RCA products fixed on the chip surface. Six different bacterial pathogens were simultaneously recognized utilizing this method. At low background signals, the synthetic oligonucleotides and genomic DNA could be detected at 0.5 pM and 0.5 pg/μL, respectively. This study successfully proved that the above method could be used to further establish and refine medical technology and could be used clinically as an additional treatment for human health.

This method affords a very significant platform for the detection of various bacteria pathogens. By taking merit of amplification of AuNP-RCA, the constitutionally high sensitivity of the SPR bio-sensor improved the limit of detection [56]. Successfully it overcame the problem of those single bacteria detection methods which were time-consuming, complex and with low efficiency. Also, more bacteria could be detected at same time. 

## 4. Aptamer Biosensor Based on RCA

In clinical medicine, a tumor marker is generally regarded as an indicator of a malignant tumor. Tumor markers are proteins, conjugated proteins, carbohydrates or peptides that are related to tumor formation and are produced by tumor tissues and cells [57,58]. In recent years, the detection of tumor markers has become possible with the continuing development of the aptamer technique. Aptamers are in vitro synthetic single-stranded DNA/RNA oligonucleotides obtained through systematic evolution of ligands by exponential enrichment (SELEX) [59,60]. Aptamers could bind with a great variety of molecules, proteins, nanomaterials, cells, and even chemicals with excellent affinity and specificity because of their three-dimensional structure. Aptamers had been used extensively in drug transfer, protein analysis, immunodiagnosis and treatment, biosensors, due to their many unique properties, such as easy synthesis, excellent affinity, high specificity, nice stability, and simple modification. A variety of novel technologies and programs based aptamers have been studied, including microfluidics [61], fluorescent [62], and electrochemical processes [63].

**Figure 3:**
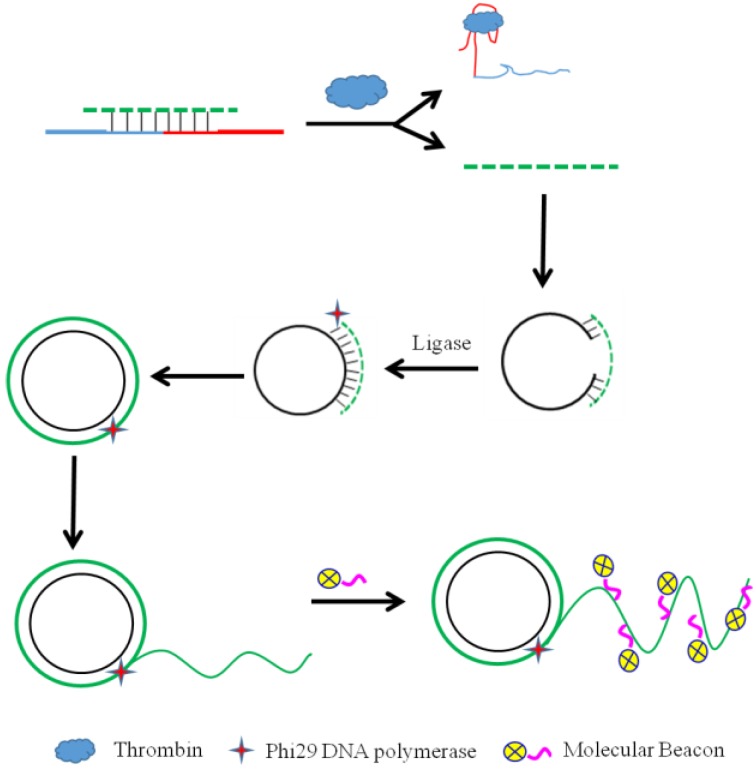
Scheme of the aptasensor design. In the lack of target molecule, the ligation-RCA step was inhibited because complementary DNA (cDNA) was hybridized to the aptamer probe to generate a double-stranded duplex. Conversely, when the target molecule was introduced, the aptamer probe bound to the target molecule with high selectivity. As a result, the cDNA hybridized with the PLP insead of the aptamer probe. The PLP was circularized by DNA T4 ligase for RCA with Phi29 DNA polymerase. The product then hybridized with the loop of molecular beacons and generated a distinct fluorescence signal [64].

As a high-efficiency isothermal amplification reaction, RCA is always coupled with biosensing methods for protein analysis. Zhang et al. [64] designed a highly sensitive fluorescent aptasensor for thrombin detection based on competition-triggered RCA All of the reactants were contained in a single reaction. In the lack of target molecule, the ligation-RCA step was inhibited because complementary DNA (cDNA) conjugated with the aptamer probe to generate a double-stranded duplex. Conversely, when the target molecule was introduced, the aptamer probe bonded to the target molecule with high selectivity. As a result, the cDNA hybridized with the PLP rather than with the aptamer probe. The PLP was circularized by DNA T4 ligase and the RCA process was achieved using Phi29 DNA polymerase. The RCA product then hybridized with the loop of molecular beacons, and generated a distinct fluorescence signal (Figure 3). The effects of the length of cDNA and concentration of PLP were studied in this work. After optimizing the operational methods, the target analyte, thrombin, was detected with high sensitivity. This method provided a new possibility for other target analytes.

Methylene blue, a biological stain, can insert into the double-stranded DNAs from the products of RCA and serves as an electrochemical label [65], allowing for label-free electrochemical sensing. For example, a label-free ultrasensitive electrochemical aptamer sensor, based on hyperbranched rolling circle amplification (HRCA) with the help of DPV, was developed by Wang et al. [66]. In their work, HRCA was coupled to an electrochemical technique to detect platelet-derived growth factor B-chain (PDGF-BB) with high sensitivity and specificity. This combined the excellent sensitivity of HRCA and the inexpensive electrochemical detection. Both the target molecule competitive reaction and the combination of electrochemical technique increased the accuracy of the result; meanwhile, the use of RCA speeded up the process of experiment. Moreover, these strategies are also applicable to other target analytes by tuning the sequence of the aptamer probe.

## 5. Micromolecular Biosensor Based on Electrochemistry Coupled to RCA

The development of electrochemical technology provides biosensors widely used in disease diagnosis and immunotherapy. RCA could produce a mass of sites on which to fasten DNA recognition probes. Owing to the otherness of binding efficiency between electroactive species and DNA, electroactive species such as tetramethylbenzidine and methylene blue are often used as electrochemical responders to enlarge the electrochemical signal [67]. Therefore, a composite of RCA and electrochemistry could be used to information output for electrochemical study.

Doxorubicin hydrochloride (DOX), a class of important anti-tumor antibiotics, produces a wide range of biochemical effects in organisms. DOX has a strong cytotoxic effect with a main mechanism of action of inhibiting nucleic acid synthesis [68]. Thus, the sensitivity of DNA sensors could be drastically enhanced using DOX as an electroactive indicator. For instance, DOX could be used to establish a high sensitivity DNA sensor [69]. An electrochemical DOX biosensor based on RCA has been established by Lu et al. [70]. In their study, DOX was intercalated into double-stranded GC or CG sequences and the DOX current signal was monitored using differential pulse voltammetry (DPV). 

**Figure 4:**
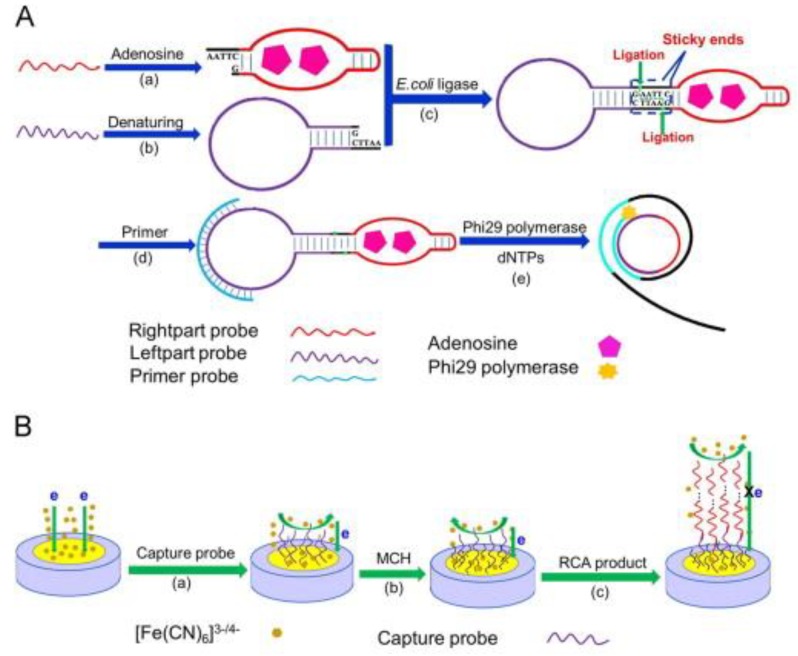
Schematic diagram of micromolecular biosensor based on electrochemistry coupled to RCA. (**A**) Formulation of RCA process based on structure-switching aptamer and sticky ends-based ligation. (a) Right part probe: target-aptamer binding, (b) left part probe: DNA denaturing to form a hairpin structure, (c) double probes were linked by *E. coli* DNA ligase with the same sticky ends, (d) the primer was adhered to the circular template by annealing, (e) RCA was initiated by adding Phi29 DNA polymerase and dNTPs. (**B**) Fabrication of the electrochemical biosensor. (a) Capture probes were anchored to the bare electrode, (b) blocking the electrode with MCH, (c) capture probe–RCA product hybridization for EIS quantitative determination [71].

Furthermore, with the help of electrochemical impedance spectroscopy, potassium ferricyanide (K_3_Fe(CN)_6)_ and DNA have been used for electrochemical signal output for ultra-sensitive detection of small molecules via ligation-RCA with analyte-mediated sticky ends (Figure 4A). In this method, 2 ss-DNA probes were designed for DNA cyclization. A “hairpin” structure was formed by the left part probe after denaturing, and right part probe also formed a similar “hairpin” structure, based on analyte-activated conformation change. Subsequently, the two probes were ligated via DNA ligase and a circular template for the RCA reaction was formed. When the adenosine was introduced, the RCA products hybridized with the capture probe on the electrode surface, prompting the impedance signal to enhance drastically, because the scheduled kinetics limit between [Fe(CN)_6_]^3−/4−^ and the negatively charged phosphate backbone of the DNA (Figure 4B). Using this electrochemical sensing technology, a fairly low detection limit and a wide linear dynamic range were accomplished by Yi et al. [71] with highly specific detection of the target.

Although these protocols are complex compared to other designs, the composite of RCA and electrochemical techniques offered a credible method for the detection of numerous small biomolecules. 

## 6. Transcription Factor (TF) Biosensor Based on RCA

A TF is a protein that is capable of binding to a specific nucleotide sequence upstream of a gene. The TF regulates the velocity of transcription of genetic information from DNA to mRNA [72] and may influence multiple transcription-associated cellular processes including cell development, differentiation, and growth [73]. TF plays a vital role in gene transcriptional regulation, and TF proteins have been confirmed to have a variety of unique features closely associated with various human diseases including cancer, abnormal hormone responses, autoimmune disorders, neurological disorders, diabetes, cardiovascular disease [74]. For example, the nuclear factor-kappaB (NF-κB) protein and the TATA-binding protein (TBP) are related to a variety of diseases [75]. Therefore, the quantification of TF proteins is essential for pharmaceutical research and immunodiagnosis.

**Figure 5:**
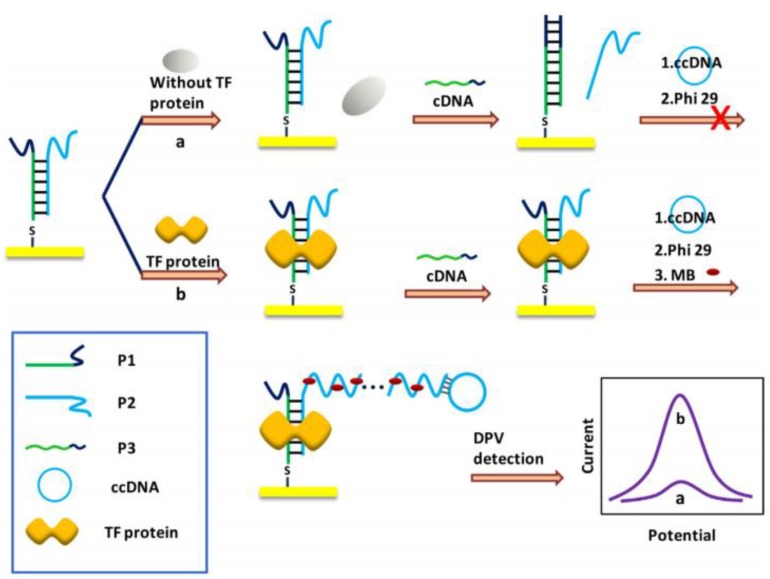
Schematic diagram of TF sensing. Three ss-DNA probes were designed for the TF biosensor. P1 was attached to the electrode surface and hybridized with P2 to form a partially complementary dsDNA. The complementary part included a TF binding sites while the non-complementary part of P2 paired with RCA primer sequence. (a) In the absence of TF protein, P3 was displaced by P2 and hybridized with P1 to form a dsDNA strand, thus stopping RCA. (b) In the presence of TF protein, a stable TF-DNA complex formed and inhibited the displacement of P3, and then, initiated the RCA with ccDNA and Phi29 DNA polymerase. Finally, the electrochemical redox probe MB was combined with the RCA product and produced the signal that was detected by DPV [33].

NF-κB p65 protein, a crucial TF, appears on multiple pathogenesis, such as inflammation, vicious circle, apoptosis, and cancer. Some corresponding measures had been developed to detect TF, such as western blot, electrophoretic mobility shift assay [76], DNase footprinting assay [77], and enzyme-linked immunosorbent assay (ELISA) [78]. However, these processes have some universal drawbacks such as insensitive, high detection limit, etc. Inspired by the advantages of RCA, Deng et al. [33] designed a highly sensitive, reproducible biosensor with good specificity for ultrasensitive TF detection. In their study, a biomedical biosensor was designed to detect TF, based on specific target molecule-DNA adsorption and RCA signal amplification. Here, three ss-DNA probes were designed for the TF biosensor. Probe 1 (P1) was attached to the electrode surface and hybridized with probe 2 (P2) to form a partially complementary dsDNA. The complementary part included a TF binding sites, while the non-complementary part of P2 paired with RCA primer sequence. Probe 3 (P3) was displaced P2 and hybridized with P1 to create a whole complementary dsDNA. In the presence of TF protein, a stable TF-DNA complex formed and initiated the RCA process. Finally, the electrochemical redox probe MB was combined with the RCA product and produced the signal that was detected by DPV (Figure 5). The fabricated biomedical sensor displayed a wide linear range and a low detection limit. 

A mutation in TBP, a kind of TF that could bond with the TATA box specifically, causes a variety of neurodegenerative diseases. Therefore, engineering a high-efficiency immunosensor has become an emerging and powerful strategy for proteome and genomics research, also for clinical diagnosis. An efficient fluorescent-amplification method for fast, quantitative, and low cost detection of TF has been developed by Li et al. [79] utilizing RCA using TBP as a target model. Three components were used in their study: TF binding probe (TFBP), enlargement primer, and dumbbell-probe. When the RCA process was complete, the products were quantized though the signal of SYBR Green I. This strategy had a very low detection limit and a wide linear region. The schematic is similar to that in Figure 5. The above two sensing strategies are simple and accurate, and they can be used for detecting other transcription factors by changing the protein-binding sequence. These protocols have advantage to be a tool for clinic diagnostic applications and high throughput bioanalysis. 

## 7. MiRNAs Biosensor Based on RCA

MicroRNAs (miRNAs) are small (18–25 nt), endogenic, non-coding, single-stranded RNA molecules that could command the expression of messenger RNA in numerous organisms [80]. Since miRNAs were found in eukaryotic cells [81], a mass of studies based on miRNA had verified that disorder in miRNA expression caused many emerging malignancies [82,83,84]. Therefore, miRNAs as novel biomarkers often be used in the diagnosis and treatment of many types of cancer [85,86,87,88], which necessitates simple, fast, reliable, sensitive, and specific tests for miRNA detection.

Traditional techniques for miRNAs detection include Northern blotting [89], RT-PCR [90], and microarrays [91]. Though these methods all have merits, they still have some drawbacks, such as low efficiency, low sensitivity, and being time-consuming. It’s necessary to select an effective amplification measure for accurate detection of miRNAs. A number of amplification strategies have been proposed, such as exonuclease (Exo)-assisted signal amplification [92,93], strand displacement amplification (SDA) [94], and RCA [95,96,97,98]. Among the many amplification methods, RCA may be the most suitable for miRNA detection because these short RNAs are optimal substrates for the ligation reaction that is needed to launch the process of RCA.

**Figure 6:**
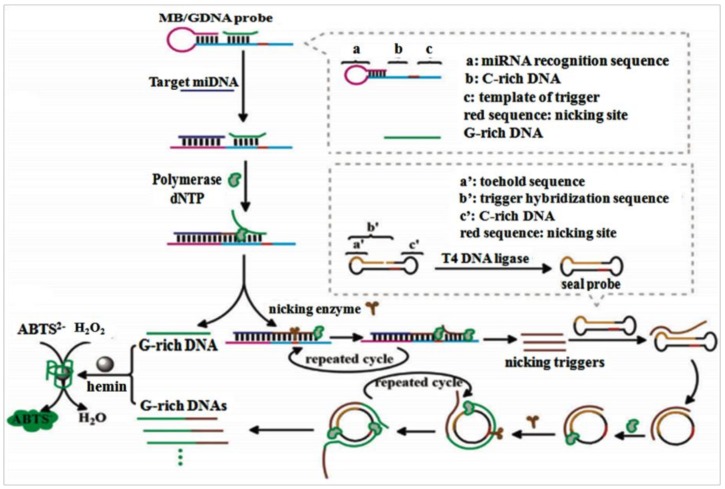
Schematic diagram of the functional nucleic acid-based amplification system for miRNA detection. The MB/ GDNA probe were composed of four domains (see right frame). The hybridization was achieved between target miRNA and domain (a); then, the target miRNA was extended to domain (b) and domain (c) to form a complete duplex by Phi29 DNA polymerase (adhered to the domain b). Then, the duplex nicking site was recognized by the nicking enzyme specifically, and the extended DNA strand was cleaved at domain (c). The TIRCA was triggered when the seal probe was annealed with the primer which were nicking triggers and released by the circulation of the extension and cleavage processes at domain (c). When the toehold domain of seal probe was bound with trigger, the spontaneous branch migration leaded to an activated circular form, and the RCA was initiated. The resultant DNA duplex was cleaved at the nicking site in the circle by nicking enzyme. Hundreds of tandem repeats of GDNA was produced as the primers were extended by Phi29 DNA polymerase. Consequently, once the presence of hemin, the colorimetric detection was amplified by the formation of DNAzyme [99].

A label-free ultrasensitive colorimetric biosensing scheme for miRNA detection had been reported by Li et al. [99]. To improve the analytical efficiency of RCA-based amplification strategies for miRNA detection, with the mutual assistance of trigger template, C-rich DNA co-modified molecular beacon (MB), G-rich DNA (GDA) probe, MB-mediated SDA, and toehold-initiated rolling circle amplification (TIRCA) to compose into a new-type, functionalized, nucleic acid-based amplification system for miRNAs detection with specificity and sensitivity (Figure 6). The amplification system consists of three parts: MB/GDNA probe, polymerase & nicking enzyme, and a seal prime (dumb-bell-shaped amplification template). MB/GDNA combined with miRNA and promoted the MB-mediated SDA to continuously produce nicking triggers, which then hybridized with the seal probe to launch TIRCA reaction and release a mountain of GDNAs. The connection of GDNAs and hemin to create a G-quadruplex/hemin DNAzyme, a famous horse radish peroxidase mimic, which catalyzed a colorimetric reaction. Notably, the 3′-OH of MB and GDNA could be deactivated by a C6 spacer, which blocked the nonspecific polymerization along with the 5′ protruding termini of MB, thus increasing the target molecule identification and decreasing the background signal. The project exhibited high sensitivity and specificity with a wide linear dynamic range, and enabled successful visual analysis the minute amount of miRNA in some real samples via the naked eye. This simple and high-efficiency signal amplification strategy built a fast and sensitive platform for miRNA detection.

Moreover, Deng’s group [97] utilized toehold-mediated strand displacement (TMSD) to design a structure-switchable seal probe to activate the RCA reaction of specific RNAs, a proposed that could accomplish the precise recognition and in situ enlargement of the target miRNA. In this miRNA course, the design of a dumbbell-shaped seal probe was the key for TMSD and RCA. The TMSD process was initiated by the target miRNA and caused a structural change in the probe. When miRNA was combined by the toehold domain, spontaneous branch migration prompted the circular form, and started RCA process.

Nevertheless, the stabilized dumbbell structure will stop the mismatched miRNA and terminated the amplification. After the seal probe was activated, the target miRNA was enlarged to lots of tandem repeat sequences by RCA and the evident diffraction-limited spot corresponding to a single miRNA will eliminate the FAM-labeled probe interference from background signal. Through this process, both stringent recognition and in situ amplification of the target miRNA can be achieved. The visualization analysis of individual miRNAs in single cells is accomplished.

## 8. Protein Biosensor Based on RCA

In recent years, with improvements in people’s standard of living, a variety of protein-related diseases have appeared. For example, gastric cancer, which was characterized by many oncoprotein biomarkers, such as p21, p27, p53, CyclinE, C-myc, Galectin-3, miR-221, MCM7, Runx3, etc. [100]. Therefore, an accurate and efficient method for protein detection is essential for disease diagnosis and clinical applications. In the traditional method of protein detection, antibodies offered a testing tool for proteins detection [101,102]; however, antibodies are generally from in vivo and cannot be synthesized in vitro. In contrast, aptamers can be synthesized in vitro through the SELEX technique. Aptamers have been widely used to fabricate biosensors in numerous new fields of study [63,103,104,105].

**Figure 7:**
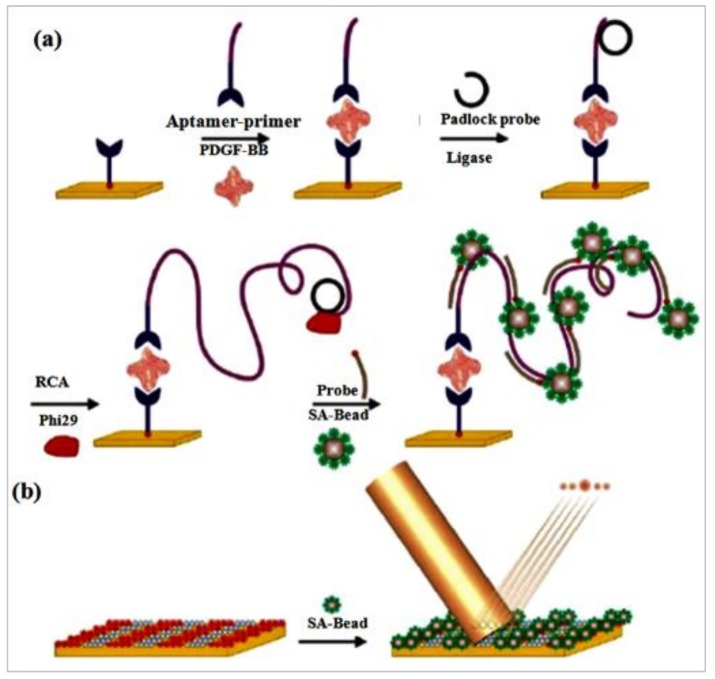
(**a**) Scheme of RCA-based microbead combines with aptamers for detection assay. Streptavidin coated periodic patterns links a biotinylated anti-PDGF-B specific aptamer. PDGF-BB was added and bound by the aptamer. An aptamer-primer complex recognized and bound to the protein. The primer tail of the aptamer was hybridized by a padlock probe, thereby initiating the RCA. The elongated concatemers hybridized with biotinylated probes which bound with streptavidin conjugated beads. (**b**) The diffraction gratings were formed by self-assembled streptavidin (SA)-coated beads on the RCA-based micropattern. The diffraction modes yielded when the illumination with a laser carried out [107].

Optical diffraction biosensors have been widely used to distinguish the binding process of many biomolecules, based on changes in effective height or refractive index on periodically patterned gratings [106]. Utilizing the many advantages of RCA, Lee et al. [107] achieved a diffraction biosensor for proteins using microbead-based RCA. In their work, aptamer and RCA were connected to detect PDGF-BB, which is related to cancer cell proliferation and transformation [108,109]. In the presence of PDGF-BB, a sandwich complex was formed between two anti-PDGF-BB aptamers (Figure 7). One of the aptamers contained a primer binding site, and the complex of aptamer and primer was used to help the product of RCA to directly localize on PDGF. The RCA reaction was initiated and the primer sequence was extended into a long oligonucleotide with the aid of phi 29 DNA polymerase. The biotinylated aptamers were fixed on the surface where periodic patterns of streptavidin were microcontact printed using a polydimethylsiloxane stamp. After the streptavidin-labeled microbeads were introduced, the diffraction grating was completed and linked with RCA concatemers on the periodic patterns. The number of microbeads on the patterns was the main determinant to generated different intensities diffraction modes with a laser light. In the absence of RCA on periodically patterned gratings, the capacity of the aptamer-based sandwich propose was executed for testing the efficiency of RCA on the periodical patterns as a signal amplification strategy, and the result proved there wasn’t any obvious bead-binding was detected, thereby reconfirming the importance of RCA.

**Figure 8:**
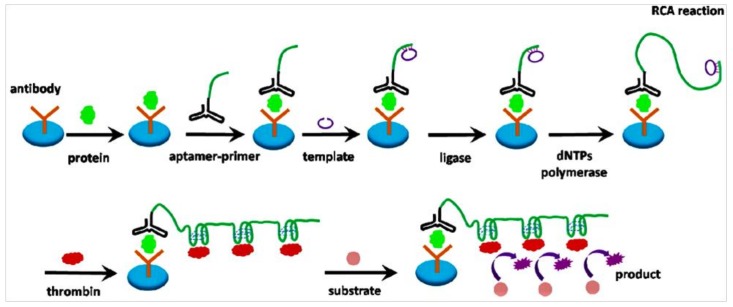
Schematic diagram of using RCA coupled with thrombin catalysis to detect protein. Microplate coated with antibody which captured the target protein. Then, the aptamer-primer complex bound with the protein. The template hybridized with the primer and was circularized by ligase, and it encoded with a complementary sequence of the aptamer for thrombin. Subsequently, the RCA initiated and a long single-stranded DNA sequence was produced for thrombin. The generated ssDNA bound with thrombin molecules, achieving multiple thrombin labeling in sandwich complex. Thrombin catalyzes small peptide substrates into detectable product [110].

The same principle, a sensitive protein biosensor, was designed by Guo et al. [110]. They used RCA coupled with thrombin catalysis to design a specific aptamer-based sandwich assay for protein detection on a microplate. This propose makes use of the long-ssDNA, with a series of tandem duplication thrombin-binding sites from RCA and the affinity of aptamer to finish various thrombin labeling and activation for signal producing (Figure 8). First, the target molecule was specifically recognized by an antibody-coated microplate. Then, a familiar sandwich structure is formed by adding aptamer and primer sequence. Following the RCA reaction was initiated to produce the combination sequence for thrombin with the help of primer. More thrombin molecules bound with these combination sequences and catalyzed the chromogenic or fluorogenic peptide substrates transformed to detectable products for final quantification of the target proteins. This protocol was used to identify PDGF-BB. With the help of two systems from RCA and thrombin catalysis, this assay had an extremely low detection limit when a fluorogenic peptide substrate was introduced. This project created a new approach for signal output in RCA relevant studies via direct thrombin labeling and evaded costing too long time on preparation of enzyme-conjugate and affinity probes.

By taking advantage of RCA, an antibody coated microplate as a signal enhancement strategy is an excellent biosensing platform. That cleverly avoided the using of complex instruments and offered a visual detection of the molecular binding events under an ordinary experiment conditions. These strategies can be used in analysis of other proteins.

## 9. Other Novel Biosensors Based on RCA

With the increasing sophistication of experimental techniques, more and more new types of immunosensors have been developed, such as sensors for the capture of oligonucleotides, proteins, cancer cells, and sensors for vitamins [111].

**Figure 9:**
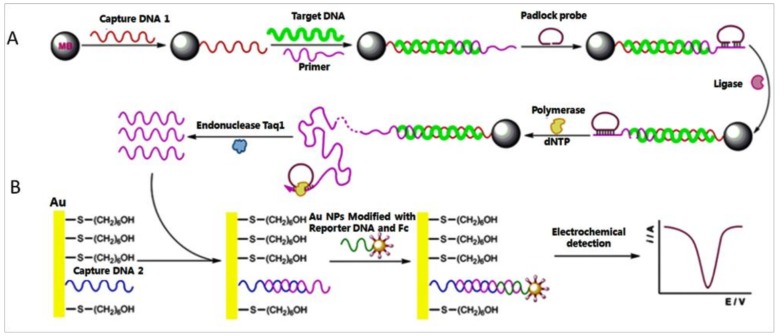
Scheme of RCA and electrochemical detection of DNA. (**A**) MBs connected with capture DNA1, and target DNA was added and tethered with it. Then, the primer DNA connected to the target DNA and the padlock probe after annealing. The RCA process was initiated to yield a long ssDNA after the padlock probe was circularized by ligase. Consequently, the long RCA products was digested to generate a crowd of short single ssDNA with same sequence as transfer DNA (t DNA) after Taq I DNA enzymes were added. (**B**) The surface of the Au electrode was covered by self-assembled DNA 2. And the electrochemical biosensor was formed by t DNA and capture DNA 2. The signal DNA loaded on Au NPs and hybridize with t DNA. The differential pulse voltammetry (DPV) scan monitored the quantity of the t DNA [116].

**Figure 10:**
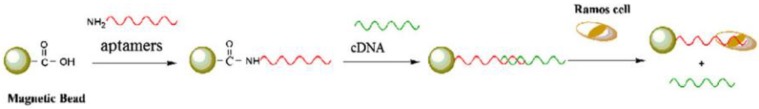
Scheme of the Ramos cells and aptamer reaction. Carboxyl-group-coated MBs connected with amino-modified aptamers of Ramos cells to form MB-DNA bio-complex which hybridized with cDNA. The bio-complex could instead of cDNA because of the stronger affinity between the aptamers and its targets when the presence of Ramos cells [116].

Nucleic acids occupy an extremely significant role in biological inheritance, and variations in them can cause immeasurable damage to an organism. Accordingly, qualitative and quantitative studies of nucleic acids contribute to the development of immunodiagnostics and pharmacogenomics [112]. Abundant methods for the detection of nucleic acids had been reported, such as SPR [113], inductively coupled plasma mass spectrometric (ICPMS) [114], chemiluminescence [115]. Nonetheless, the detection limit for DNA when using these existing scheme is still a major obstacle. Therefore, designing a novel detection method for nucleic acids is imperative. A new style of biosensor, based on RCA and nanoparticle aggregation, for ultrasensitive detection of DNA and tumor cells was developed by Ding et al. [116]. Their work included three primary steps, which are summarized as follows: first, the surface of magnetic beads was immobilized with a “sandwich-type” DNA complex contained target DNA. Then the primer in the “sandwich-type” DNA complex initiated RCA in the system. The new “sandwich-type” DNA complex were formed in the long RCA products for electrochemical detection (Figure 9). With the help of DPV, an extremely low detection limit of target DNA was obtained. Assisted with aptamer technique, this signal output strategy was also used to identify tumor cells, in which an extremely low detection limit was also achieved (Figure 10).

**Figure 11:**
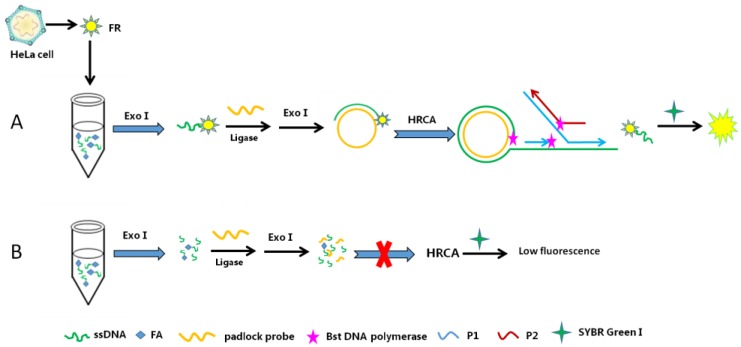
Schematic of a fluorescence biosensor for FR determination, based on HRCA and terminal protection. (**A**) FR connected with FA-ssDNA through FR-FA interaction and prevented the hydrolyzation of Exo I. Then, the compound hybridized with padlock probe, and formed a circular padlock probe with the aid of DNA ligase. Later, the HRCA reaction was activated by Bst DNA polymerase, P1, P2, and dNTPs to launch chain extensions and strand displacements, resulting a mass of long double-strand DNA (dsDNA) and ssDNA yielded. High fluorescence signal was monitored after introducing SYBR Green I. (**B**) In the absence of the target, ssDNA were hydrolyzed by Exo I and the HRCA was inhibited, so only weak fluorescence signal was detected [118].

Folic acid (FA), a water-soluble vitamin, promotes the maturation of bone marrow cells in the human. A deficiency in FA can cause megaloblastic anemia and leucopenia, which is particularly important for pregnant women. Therefore, monitoring the FA level is vital for the immunodiagnosis and treatment of cancers and chronic inflammatory diseases [117]. For example, a fluorescence biosensor for folate receptor (FR) in cancer cells based on terminal protection and HRCA was developed by Li et al. [118]. In their work, ssDNA terminally tethered to FA specifically combined with FR and was protected from digestion by exonuclease I (Exo I). The protected ssDNA hybridized with the padlock probe and triggered the HRCA reaction. Conversely, the ssDNA was digested by Exo I and no probe was left to initiate the HRCA reaction without the protection of the target FR. In the presence of SYBR Green I, the HRCA products included a mass of double-stranded DNA and a strong fluorescence signal could be detected (Figure 11). Moreover, the developed biosensor is also used to detect FR in cancer cells (e.g., HeLa cells). The increased fluorescence intensity has shown lower detection limit with a wide linear dynamic range.

This protocol offered a simple, fast and efficient method for the identification of FR. At the same time, it effectively prevented the ssDNA from hydrolysis by Exo I, and made full use of the advantages of HRCA rapid expansion, which not only shortened the working time, but also increased the reliability of the result. 

## 10. Outlook

RCA is an excellent signal amplification technology. It is a highly versatile DNA amplification tool widely used in many fields where limitations in sensitivity and/or specificity, laborious sample preparation and/or signal amplification procedures had previously hindered the use of other tools. RCA is used for assays and procedures in fields such as immunohistochemistry, immunodiagnostics, nanotechnology, materials science, genomics, proteomics, biosensing, drug discovery, targeted therapy, flow cytometry. Moreover, the combination of RCA with functional nucleic acids, containing aptamers and DNAzymes, and with other assay platforms involve PCR, SELEX, optical diffraction, ELISA, microfluidics, ICPMS, chemiluminescence, SPR, and nanoparticles, presents new opportunities for ultrasensitive detection of a lot of targets, including nucleic acids, proteins, vitamins, small molecules, viruses, hormones, and cells. Particularly, RCA has increased the sensitivity of medical detection and greatly reduced the detection limit. In recent years, the combination of RCA with aptamers and nanomaterials has attracted more attention. Integrating these three technologies will greatly promote human health and medicine. As the standard of living continues to increase, more new diseases will likely emerge, necessitating continued research to ensure human health and social development. The combination of new generations of biosensors with RCA and/or other technologies holds great potential for further applications in biomedical research and early clinical diagnostics.

## References

[1] Nilsson M., Gullberg M., Dahl F., Szuhai K., Raap A.K. (2002). Real-time monitoring of rolling-circle amplification using a modified molecular beacon design. Nucleic Acids Res..

[2] Liu X., Xue Q., Ding Y., Zhu J., Wang L., Jiang W. (2014). Cascade Signal Amplification Strategy for Sensitive and Label-free DNA Detection Based on Exo III-catalyzed Recycling Coupled with Rolling Circle Amplification. Analyst.

[3] Li Y., Zeng Y., Ji X., Li X., Ren R. (2012). Cascade signal amplification for sensitive detection of cancer cell based on self-assembly of DNA scaffold and rolling circle amplification. Sens. Actuators B Chem..

[4] Khan S.A. (2005). Plasmid rolling-circle replication: Highlights of two decades of research. Plasmid.

[5] Zhao W., Cui C.H., Bose S., Guo D., Shen C., Wong W.P., Halvorsen K., Farokhzad O.C., Teo G.S.L., Phillips J.A. (2012). Bioinspired multivalent DNA network for capture and release of cells. Proc. Natl. Acad. Sci. USA.

[6] Zhang Z., Ali M.M., Eckert M.A., Kang D.K., Chen Y.Y., Sender L.S., Fruman D.A., Zhao W. (2013). A polyvalent aptamer system for targeted drug delivery. Biomaterials.

[7] Ali M.M., Li Y. (2009). Colorimetric Sensing by Using Allosteric-DNAzyme-Coupled Rolling Circle Amplification and a Peptide Nucleic Acid–Organic Dye Probe. Angew. Chem..

[8] Cheglakov Z., Weizmann Y., Basnar B., Willner I. (2007). Diagnosing viruses by the rolling circle amplified synthesis of DNAzymes. Org. Biomol. Chem..

[9] Dong H., Wang C., Xiong Y., Lu H., Ju H., Zhang X. (2013). Highly sensitive and selective chemiluminescent imaging for DNA detection by ligation-mediated rolling circle amplified synthesis of DNAzyme. Biosens. Bioelectron..

[10] Tang L., Liu Y., Ali M.M., Kang D.K., Zhao W., Li J. (2012). Colorimetric and ultrasensitive bioassay based on a dual-amplification system using aptamer and DNAzyme. Anal. Chem..

[11] Dahl F., Banér J., Gullberg M., Mendel-Hartvig M., Landegren U., Nilsson M. (2004). Circle-to-circle amplification for precise and sensitive DNA analysis. Proc. Natl. Acad. Sci. USA.

[12] Linck L., Reiß E., Bier F., Resch-Genger U. (2012). Direct labeling rolling circle amplification as a straightforward signal amplification technique for biodetection formats. Anal. Methods.

[13] Zhao W., Gao Y., Kandadai S.A., Brook M.A., Li Y. (2006). DNA Polymerization on Gold Nanoparticles through Rolling Circle Amplification: Towards Novel Scaffolds for Three-Dimensional Periodic Nanoassemblies. Angew. Chem. Int. Ed..

[14] Ali M.M., Aguirre S.D., Xu Y., Filipe C.D.M., Pelton R., Li Y. (2009). Detection of DNA using bioactive paper strips. Chem. Commun..

[15] Berr A., Schubert I. (2007). Interphase chromosome arrangement in Arabidopsis thaliana is similar in differentiated and meristematic tissues and shows a transient mirror symmetry after nuclear division. Genetics.

[16] Beyer S., Nickels P., Simmel F.C. (2005). Periodic DNA nanotemplates synthesized by rolling circle amplification. Nano Lett..

[17] Su H., Yuan R., Chai Y., Mao L., Zhuo Y. (2011). Ferrocenemonocarboxylic–HRP@ Pt nanoparticles labeled RCA for multiple amplification of electro-immunosensing. Biosens. Bioelectron..

[18] Thomas D., Lizardi P., Nardone G., Winndeen E. (1997). Cascade rolling circle amplification, a homogeneous fluorescence detection system for DNA diagnostics. Clin. Chem..

[19] Smolina I., Lee C., Frank-Kamenetskii M. (2007). Detection of low-copy-number genomic DNA sequences in individual bacterial cells by using peptide nucleic acid-assisted rolling-circle amplification and fluorescence in situ hybridization. Appl. Environ. Microbiol..

[20] Xu W., Xie X., Li D., Yang Z., Li T., Liu X. (2012). Ultrasensitive Colorimetric DNA Detection using a Combination of Rolling Circle Amplification and Nicking Endonuclease-Assisted Nanoparticle Amplification (NEANA). Small.

[21] Johne R., Müller H., Rector A., Van Ranst M., Stevens H. (2009). Rolling-circle amplification of viral DNA genomes using phi29 polymerase. Trends Microbiol..

[22] Schopf E., Fischer O.N., Chen Y., Tok J.B.H. (2008). Sensitive and selective viral DNA detection assay via microbead-based rolling circle amplification. Bioorg. Med. Chem. Lett..

[23] Schopf E., Chen Y. (2010). Attomole DNA detection assay via rolling circle amplification and single molecule detection. Anal. Biochem..

[24] Thomas D.C., Nardone G.A., Randall S.K. (1999). Amplification of padlock probes for DNA diagnostics by cascade rolling circle amplification or the polymerase chain reaction. Arch. Pathol. Lab. Med..

[25] Christian A.T., Pattee M.S., Attix C.M., Reed B.E., Sorensen K.J., Tucker J.D. (2001). Detection of DNA point mutations and mRNA expression levels by rolling circle amplification in individual cells. Proc. Natl. Acad. Sci. USA.

[26] Lagunavicius A., Merkiene E., Kiveryte Z., Savaneviciute A., Zimbaite-Ruskuliene V., Radzvilavicius T., Janulaitis A. (2009). Novel application of Phi29 DNA polymerase: RNA detection and analysis in vitro and in situ by target RNA-primed RCA. RNA.

[27] Zhou Y., Calciano M., Hamann S., Leamon J.H., Strugnell T., Christian M.W., Lizardi P.M. (2001). In situ detection of messenger RNA using digoxigenin-labeled oligonucleotides and rolling circle amplification. Exp. Mol. Pathol..

[28] Zhao H., Ma X., Li M., Zhou D., Xiao P., Lu Z. (2011). Analysis of CpG island methylation using rolling circle amplification (RCA) product microarray. J. Biomed. Nanotechnol..

[29] Qi X., Bakht S., Devos K.M., Gale M.D., Osbourn A. (2001). L-RCA (ligation-rolling circle amplification): A general method for genotyping of single nucleotide polymorphisms (SNPs). Nucleic Acids Res..

[30] Pickering J., Bamford A., Godbole V., Briggs J., Scozzafava G., Roe P., Wheeler C., Ghouze F., Cuss S. (2002). Integration of DNA ligation and rolling circle amplification for the homogeneous, end-point detection of single nucleotide polymorphisms. Nucleic Acids Res..

[31] Tang Z.Y., Cheng Y.Q., Du Q., Zhang H.X., Li Z.P. (2011). Integration of rolling circle amplification and cationic conjugated polymer for the homogeneous detection of single nucleotide polymorphisms. Chin. Sci. Bull..

[32] Zhang S., Wu Z., Shen G., Yu R. (2009). A label-free strategy for SNP detection with high fidelity and sensitivity based on ligation-rolling circle amplification and intercalating of methylene blue. Biosens. Bioelectron..

[33] Deng K., Li C., Huang H., Li X. (2017). Rolling circle amplification based on signal-enhanced electrochemical DNA sensor for ultrasensitive transcription factor detection. Sens. Actuators B Chem..

[34] Meng F., Miao P., Wang B., Tang Y., Yin J. (2016). Identification of glutathione by voltammetric analysis with rolling circle amplification. Anal. Chim. Acta.

[35] Shi H., Mao X., Chen X., Wang Z., Wang K., Zhu X. (2017). The analysis of proteins and small molecules based on sterically tunable nucleic acid hyperbranched rolling circle amplification. Biosens. Bioelectron..

[36] Ding C., Liu H., Wang N., Wang Z. (2012). Cascade signal amplification strategy for the detection of cancer cells by rolling circle amplification and nanoparticles tagging. Chem. Commun..

[37] Maruyama F., Kenzaka T., Yamaguchi N., Tani K., Nasu M. (2005). Visualization and enumeration of bacteria carrying a specific gene sequence by in situ rolling circle amplification. Appl. Environ. Microbiol..

[38] Ali M.M., Li F., Zhang Z., Zhang K., Kang D.K., Ankrum J.A., Chris Le X., Zhao W. (2014). Rolling circle amplification: A versatile tool for chemical biology, materials science and medicine. Chem. Soc. Rev..

[39] Doherty A.J., Dafforn T.R. (2000). Nick Recognition by DNA Ligases. J. Mol. Biol..

[40] Doherty A.J., Suh S.W. (2000). Structural and mechanistic conservation in DNA ligases. Nucleic Acids Res..

[41] Lehman I. (1974). DNA Iigase: Structure mechanism, function. Science.

[42] Blundred R.M., Stewart G.S. (2011). DNA double-strand break repair, immunodeficiency and the RIDDLE syndrome. Expert Rev. Clin. Immunol..

[43] Yu J.-C., Ding S., Chang C.H., Kuo S.H., Chan S.T., Hsu G.C., Hsu H.M., Hou M.F., Jung L.Y., Cheng C.W. (2009). Genetic susceptibility to the development and progression of breast cancer associated with polymorphism of cell cycle and ubiquitin ligase genes. Carcinogenesis.

[44] Siribal S., Weinfeld M., Karimi-Busheri F., Mark Glover J.N., Bernstein N.K., Aceytuno D., Chavalitshewinkoon-Petmitr P. (2011). Molecular characterization of Plasmodium falciparum putative polynucleotide kinase/phosphatase. Mol. Biochem. Parasitol..

[45] Allinson S.L. (2010). DNA end-processing enzyme polynucleotide kinase as a potential target in the treatment of cancer. Future Oncol..

[46] Freschauf G.K., Karimi-Busheri F., Ulaczyk-Lesanko A., Mereniuk T.R., Ahrens A., Koshy J.M., Rasouli-Nia A., Pasarj P., Holmes C.F.B., Rininsland F. (2009). Identification of a small molecule inhibitor of the human DNA repair enzyme polynucleotide kinase/phosphatase. Cancer Res..

[47] Jiang H.-X., Kong D.-M., Shen H.-X. (2014). Amplified detection of DNA ligase and polynucleotide kinase/phosphatase on the basis of enrichment of catalytic G-quadruplex DNAzyme by rolling circle amplification. Biosens. Bioelectron..

[48] Bodrossy L., Sessitsch A. (2004). Oligonucleotide microarrays in microbial diagnostics. Curr. Opin. Microbiol..

[49] Eriksson R., Jobs M., Ekstrand C., Ullberg M., Herrmann B., Landegren U., Nilsson M., Blomberg J. (2009). Multiplex and quantifiable detection of nucleic acid from pathogenic fungi using padlock probes, generic real time PCR and specific suspension array readout. J. Microbiol. Methods.

[50] Atkins S.D., Clark I.M. (2004). Fungal molecular diagnostics: A mini review. J. Appl. Genet..

[51] Cho S.-N., Brennan P.J. (2007). Tuberculosis: Diagnostics. Tuberculosis.

[52] López M.M., Bertolini E., Olmos A., Caruso P., Gorris M.T., Llop P., Penyalver R., Cambra M. (2003). Innovative tools for detection of plant pathogenic viruses and bacteria. Int. Microbiol..

[53] Manso J., Mena M.L., Yanez-Sedeno P., Pingarrón J.M. (2008). Bienzyme amperometric biosensor using gold nanoparticle-modified electrodes for the determination of inulin in foods. Anal. Biochem..

[54] Homola J. (2008). Surface plasmon resonance sensors for detection of chemical and biological species. Chem. Rev..

[55] Cosnier S., Mailley P. (2008). Recent advances in DNA sensors. Analyst.

[56] Shi D., Huang J., Chuai Z., Chen D., Zhu X., Wang H., Peng J., Wu H., Huang Q., Fu W. (2014). Isothermal and rapid detection of pathogenic microorganisms using a nano-rolling circle amplification-surface plasmon resonance biosensor. Biosens. Bioelectron..

[57] Daniel D., Lalitha R. (2016). Tumor markers—A bird’s eye view. J. Oral Maxillofac. Surg. Med. Pathol..

[58] De Rancher M.-A.R., Oudart J.B., Maquart F.X., Monboisse J.C., Ramont L. (2016). Evaluation of Lumipulse^®^ G1200 for the measurement of six tumor markers: Comparison with AIA^®^ 2000. Clin. Biochem..

[59] Chung S., Moon J.M., Ban C., Shim Y.B. (2016). A Simple and Fast SELEX Using an Alternating Current Potential Modulated Microfluidic Channel and an Evaluation of Sensing Ability of Aptamers. Meeting Abstracts.

[60] Mu Q., Annapragada A., Srivastava M., Thiviyanathan V., Li X., Gorenstein D., Annapragada A., Vigneswaran N. (2016). Conjugate-SELEX, a novel screening method, identifies aptamers that deliver payload to the cytosol of target cells. Cancer Res..

[61] Sanghavi B.J., Moore J.A., Chávez J.L., Hagen J.A., Kelley-Loughnane N., Chou C.F., Swami N.S. (2016). Aptamer-functionalized nanoparticles for surface immobilization-free electrochemical detection of cortisol in a microfluidic device. Biosens. Bioelectron..

[62] Théodorou I., Quang N.N., Gombert K., Thézé B., Lelandais B., Ducongé F. (2016). In Vitro and In Vivo Imaging of Fluorescent Aptamers. Nucleic Acid Aptamers: Selection, Characterization, and Application.

[63] Wu L., Qi P., Fu X., Liu H., Li J., Wang Q., Fan H. (2016). A novel electrochemical PCB77-binding DNA aptamer biosensor for selective detection of PCB77. J. Electroanal. Chem..

[64] Zhang S.-B., Zheng L.Y., Hu X., Shen G.Y., Liu X.W., Shen G.L., Yu R.Q. (2015). Highly Sensitive Fluorescent Aptasensor for Thrombin Detection Based on Competition Triggered Rolling Circle Amplification. Chin. J. Anal. Chem..

[65] Kerman K., Ozkan D., Kara P., Meric B., Gooding J.J., Ozsoz M. (2002). Voltammetric determination of DNA hybridization using methylene blue and self-assembled alkanethiol monolayer on gold electrodes. Anal. Chim. Acta.

[66] Wang Q., Zheng H., Gao X., Lin Z., Chen G. (2013). A label-free ultrasensitive electrochemical aptameric recognition system for protein assay based on hyperbranched rolling circle amplification. Chem. Commun..

[67] Alves-Balvedi R.P., Caetano L.P., Madurro J.M., Brito-Madurro A.G. (2016). Use of 3,3′,5,5′ tetramethylbenzidine as new electrochemical indicator of DNA hybridization and its application in genossensor. Biosens. Bioelectron..

[68] Berg H., Horn G., Luthardt U., Ihn W. (1981). Interaction of anthracycline antibiotics with biopolymers: Part V. Polarographic behavior and complexes with DNA. Bioelectrochem. Bioenerget..

[69] Zhang Y., Zhang K., Ma H. (2009). Electrochemical DNA biosensor based on silver nanoparticles/poly (3-(3-pyridyl) acrylic acid)/carbon nanotubes modified electrode. Anal. Biochem..

[70] Lu L., Liu B., Zhao Z., Ma C., Luo P., Liu C., Xie G. (2012). Ultrasensitive electrochemical immunosensor for HE4 based on rolling circle amplification. Biosens. Bioelectron..

[71] Yi X., Li L., Peng Y., Guo L. (2014). A universal electrochemical sensing system for small biomolecules using target-mediated sticky ends-based ligation-rolling circle amplification. Biosens. Bioelectron..

[72] Latchman D.S. (1997). Transcription factors: An overview. Int. J. Biochem. Cell Biol..

[73] Rosenbauer F., Tenen D.G. (2007). Transcription factors in myeloid development: Balancing differentiation with transformation. Nat. Rev. Immunol..

[74] Engelkamp D., van Heyningen V. (1996). Transcription factors in disease. Curr. Opin. Genet. Dev..

[75] Zhang Y., Ma F., Tang B., Zhang C. (2016). Recent advances in transcription factor assays in vitro. Chem. Commun..

[76] Garner M.M., Revzin A. (1981). A gel electrophoresis method for quantifying the binding of proteins to specific DNA regions: Application to components of the *Escherichia coli* lactose operon regulatory system. Nucleic Acids Res..

[77] Galas D.J., Schmitz A. (1978). DNAase footprinting a simple method for the detection of protein-DNA binding specificity. Nucleic Acids Res..

[78] Burnette W.N. (1981). “Western blotting”: Electrophoretic transfer of proteins from sodium dodecyl sulfate-polyacrylamide gels to unmodified nitrocellulose and radiographic detection with antibody and radioiodinated protein A. Anal. Biochem..

[79] Li C., Qiu X., Hou Z., Deng K. (2015). A dumbell probe-mediated rolling circle amplification strategy for highly sensitive transcription factor detection. Biosens. Bioelectron..

[80] Liang Y., Ridzon D., Wong L., Chen C. (2007). Characterization of microRNA expression profiles in normal human tissues. BMC Genom..

[81] Ambros V. (2004). The functions of animal microRNAs. Nature.

[82] Heneghan H.M., Miller N., Lowery A.J., Sweeney K.J., Newell J., Kerin M.J. (2010). Circulating microRNAs as novel minimally invasive biomarkers for breast cancer. Ann. Surg..

[83] Li C., Feng Y., Coukos G., Zhang L. (2009). Therapeutic microRNA strategies in human cancer. AAPS J..

[84] Zhu W., Qin W., Atasoy U., Sauter E.R. (2009). Circulating microRNAs in breast cancer and healthy subjects. BMC Res. Notes.

[85] Stenvang J., Silahtaroglu A.N., Lindow M., Elmen J., Kauppinen S. (2008). The utility of LNA in microRNA-based cancer diagnostics and therapeutics. Seminars in Cancer Biology.

[86] Cho W.C. (2010). MicroRNAs: Potential biomarkers for cancer diagnosis, prognosis and targets for therapy. Int. J. Biochem. Cell Biol..

[87] Li J., Tan S., Kooger R., Zhang C., Zhang Y. (2014). MicroRNAs as novel biological targets for detection and regulation. Chem. Soc. Rev..

[88] Volinia S., Calin G.A., Liu C.G., Ambs S., Cimmino A., Petrocca F., Visone R., Iorio M., Roldo C., Ferracin M. (2006). A microRNA expression signature of human solid tumors defines cancer gene targets. Proc. Natl. Acad. Sci. USA.

[89] Válóczi A., Hornyik C., Varga N., Burgyán J., Kauppinen S., Havelda Z. (2004). Sensitive and specific detection of microRNAs by northern blot analysis using LNA-modified oligonucleotide probes. Nucleic Acids Res..

[90] Chen C., Ridzon D.A., Broomer A.J., Zhou Z., Lee D.H., Nguyen J.T., Barbisin M., Xu N.L., Mahuvakar V.R., Andersen M.R. (2005). Real-time quantification of microRNAs by stem–loop RT–PCR. Nucleic Acids Res..

[91] Li W., Ruan K. (2009). MicroRNA detection by microarray. Anal. Bioanal. Chem..

[92] Bi S., Li L., Cui Y. (2012). Exonuclease-assisted cascaded recycling amplification for label-free detection of DNA. Chem. Commun..

[93] Wang M., Fu Z., Li B., Zhou Y., Yin H., Ai S. (2014). One-step, ultrasensitive, and electrochemical assay of microRNAs based on T7 exonuclease assisted cyclic enzymatic amplification. Anal. Chem..

[94] Liu Y.Q., Zhang M., Yin B.C., Ye B.C. (2012). Attomolar ultrasensitive microRNA detection by DNA-scaffolded silver-nanocluster probe based on isothermal amplification. Anal. Chem..

[95] Bi S., Cui Y., Dong Y., Zhang N. (2014). Target-induced self-assembly of DNA nanomachine on magnetic particle for multi-amplified biosensing of nucleic acid, protein, and cancer cell. Biosens. Bioelectron..

[96] Liu H., Li L., Duan L., Wang X., Xie Y., Tong L., Wang Q., Tang B. (2013). High specific and ultrasensitive isothermal detection of microRNA by padlock probe-based exponential rolling circle amplification. Anal. Chem..

[97] Deng R., Tang L., Tian Q., Wang Y., Lin L., Li J. (2014). Toehold-initiated Rolling Circle Amplification for Visualizing Individual MicroRNAs In Situ in Single Cells. Angew. Chem. Int. Ed..

[98] JamesáYang C. (2014). A T7 exonuclease-assisted cyclic enzymatic amplification method coupled with rolling circle amplification: A dual-amplification strategy for sensitive and selective microRNA detection. Chem. Commun..

[99] Li D., Cheng W., Yan Y., Zhang Y., Yin Y., Ju H., Ding S. (2016). A colorimetric biosensor for detection of attomolar microRNA with a functional nucleic acid-based amplification machine. Talanta.

[100] Gao X. (2016). The Progress Study of Gastric Cancer and Tumor Associated Protein. Asian Case Rep. Oncol..

[101] Olmsted J. (1981). Affinity purification of antibodies from diazotized paper blots of heterogeneous protein samples. J. Biol. Chem..

[102] Stavitsky A.B., Jarchow C. (1954). Micromethods for the study of proteins and antibodies I. Procedure and general applications of hemagglutination and hemagglutination-inhibition reactions with tannic acid and protein-treated red blood cells. J. Immunol..

[103] Savran C.A., Knudsen S.M., Ellington A.D., Manalis S.R. (2004). Micromechanical detection of proteins using aptamer-based receptor molecules. Anal. Chem..

[104] Eissa S., Ng A., Siaj M., Zourob M. (2015). Selection, Characterization, and Application of High Affinity Microcystin-Targeting Aptamers in a Graphene-Based Biosensing Platform. Meeting Abstracts.

[105] Chen I.H., Horikawa S., Du S., Liu Y., Wikle H.C., Barbaree J.M., Chin B.A. (2016). Thermal Stability of Phage Peptide Probes vs. Aptamer for Salmonella Detection on Magnetoelastic Biosensors Platform. ECS Trans..

[106] Acharya G., Chang C.L., Doorneweerd D.D., Vlashi E., Henne W.A., Hartmann L.C., Low P.S., Savran C.A. (2007). Immunomagnetic diffractometry for detection of diagnostic serum markers. J. Am. Chem. Soc..

[107] Lee J., Icoz K., Roberts A., Ellington A.D., Savran C.A. (2010). Diffractometric detection of proteins using microbead-based rolling circle amplification. Anal. Chem..

[108] Noskovicova N., Petřek M., Eickelberg O., Heinzelmann K. (2015). Platelet-derived growth factor signaling in the lung. From lung development and disease to clinical studies. Am. J. Respir. Cell Mol. Biol..

[109] Kuai J., Mosyak L., Brooks J., Cain M., Carven G.J., Ogawa S., Ishino T., Tam M., Lavallie E.R., Yang Z. (2015). Characterization of binding mode of action of a blocking anti-platelet-derived growth factor (PDGF)-B monoclonal antibody, MOR8457, reveals conformational flexibility and avidity needed for PDGF-BB to bind PDGF receptor-β. Biochemistry.

[110] Guo L., Hao L., Zhao Q. (2016). An aptamer assay using rolling circle amplification coupled with thrombin catalysis for protein detection. Anal. Bioanal. Chem..

[111] Jiang W., Liu L., Zhang L., Guo Q., Cui Y., Yang M. (2017). Sensitive immunosensing of the carcinoembryonic antigen utilizing aptamer-based in-situ formation of a redox-active heteropolyacid and rolling circle amplification. Microchim. Acta.

[112] Risch N., Merikangas K. (1996). The future of genetic studies of complex human diseases. Science.

[113] Fang Y., Orner B.P. (2006). Induction of pluripotency in fibroblasts through the expression of only four nuclear proteins. ACS Chem. Biol..

[114] Zhang J., Chua L.S., Lynn D.M. (2004). Multilayered thin films that sustain the release of functional DNA under physiological conditions. Langmuir.

[115] Miao W., Bard A.J. (2004). Electrogenerated chemiluminescence. 77. DNA hybridization detection at high amplification with [Ru(bpy)3]^2+^-containing microspheres. Anal. Chem..

[116] Ding C., Wang N., Zhang J., Wang Z. (2013). Rolling circle amplification combined with nanoparticle aggregates for highly sensitive identification of DNA and cancercells. Biosens. Bioelectron..

[117] Akbar S., Anwar A., Kanwal Q. (2016). Electrochemical determination of folic acid: A short review. Anal. Biochem..

[118] Li D., Ma Y., Zhang Y., Lin Z. (2016). Fluorescence biosensor for folate receptors in cancer cells based on terminal protection and hyperbranched rolling circle amplification. Anal. Methods.

